# Phase angle and its determinants among adolescents: influence of body composition and physical fitness level

**DOI:** 10.1038/s41598-024-62546-6

**Published:** 2024-06-13

**Authors:** Giovana O. C. Ferreira, Gerson Ferrari, Raquel D. Langer, Marco Cossio-Bolaños, Rossana Gomez-Campos, Evandro Lázari, Anderson M. Moraes

**Affiliations:** 1 Department of Sports Science, Pontifical Catholic University of Campinas, Campinas, Brazil; 2 Escuela de Ciencias de la Actividad Física, el Deporte y la Salud, Universidad de Santiago de Chile (USACH), Santiago, Chile; 3 Facultad de Ciencias de la Salud, Universidad Autónoma de Chile, Santiago, Chile; 4 School of Medical Sciences, Growth and Development Laboratory – Center for Investigation in Pediatrics (CIPED), University of Campinas, Campinas, Brazil; 5 Universidad Católica del Maule, Talca, Chile; 6 School of Applied Sciences, University of Campinas, Campinas, Brazil; 7 School of Physical Education, Pontifical Catholic University of Campinas, Rua Prof. Dr. Euryclides De Jesus Zerbini, 1516, Campinas, 13083-9 Brazil

**Keywords:** Phase angle, Physical fitness, Body composition, Cardiorespiratory fitness, Public health, Quality of life

## Abstract

To examine the association between levels of physical fitness, parameters of body composition and phase angle (PhA) amongst adolescents. A total of 152 adolescents (84 girls) aged 11–16 years were included in this study. Weight and height were measured and the body mass index (BMI) was calculated. Bioelectrical impedance analysis (BIA) provided resistance and reactance parameters to calculate fat-free mass (FFM), PhA and fat mass (%FM). The following physical fitness variables were analysed: flexibility, abdominal muscular endurance, upper and lower limb explosive strength, agility, speed and cardiorespiratory fitness. Generalized Linear Models were applied to verify differences across sexes. Stepwise linear regression was used to establish an association between the variables studied. The study established an association between PhA and weight, FFM, BMI, FM, %FM and medicine ball throw (MBT) for girls. As for the boys, an association was verified between PhA and weight, FFM, BMI, standing long jump (SLJ), MBT and the three allometric VO_2peak_ variables analyzed_._ An association was found between PhA and the boys’ 4-m shuttle run test (4SRT) and 20-m sprint test (20SRT). Boys showed a greater phase angle than girls; In girls, BMI and %FM, were determinant of 32.4% (r = 0.57). PhA variability which is influenced by physical fitness, body composition and, therefore, the tissues electrical conductivity. Furthermore, boys’ height, FFM, upper limb strength, and agility account for 58.4% (r = 0.76) PhA variability. There was a positive correlation between the physical fitness tests and the PhA.

## Background

Being physically active and having good levels of body composition parameters [low fat mass (FM) and high fat-free mass (FFM)] are widely known as components of a healthy life style^1,2^. However, physical inactivity is currently identified as the fourth main risk factor for all-cause mortality, and, as a consequence, the increase of chronic non-communicable diseases (NCDs) has modified the population’s general health globally^1,2^.

On the other hand, previous studies have shown that children and adolescents who engage in moderate-to-vigorous physical activity one or more hours per day, and muscle and bone-strengthening activities three or more times per week with the correct load applied, have improved their muscular strength, cardiorespiratory fitness and body composition, therefore decreasing the liability of cardiovascular complications and benefitting bone health, psychological well-being, cognition and school performance^1,3,4^. In addition, developing and adopting healthy behaviors during this stage of life might reinforce well-being and health throughout adulthood^5,6^.

However, nowadays some tendencies of physical inactivity can be seen, especially concerning the youth which maintain minor levels of physical fitness than children from previous generations^7^, leading to the rise of obesity^8^, cardiovascular diseases, diabetes, hypertension, and cancer^9,10^. A previous study showed that 6% of cardiovascular diseases and 7% of diabetes type II are expected to develop worldwide due to physical inactivity^11^.

Therefore, using accurate methods to monitor physical fitness and body composition changes occurring during growth might contribute to decelerating the epidemiological risk factors associated with physical inactivity^12^.

Bioelectrical impedance analysis (BIA) is a worldwide technique used to analyze body composition in individuals with different characteristics (i.e., age, sex, ethnicity, BMI, health condition). BIA provides raw parameters of reactance (Xc) and resistance (R) in ohms (Ω). Xc is the capacitance produced by tissue interfaces and cell membranes, while R is the pure opposition of a biological conductor to the flow of an alternating electric current^13–15^. Considering that in biological systems the electrical current is conducted by the electrolytes content in the body water, the lean tissue is a good electric conductor due to the high amount of water, thus offering lower resistance to the electrical current. On the other hand, fat mass and bone mass are poorly conductive; hence have a higher resistance value^13,14,16^.

It is possible to calculate the phase angle (PhA) in degrees using BIA parameters. PhA is expressed by the arc tangent of the reactance and resistance and is used as an indicator of nutritional status in different patients, such as malnourished children and patients undergoing gastrointestinal surgery^17,18^.

Previous studies have found a direct association between PhA and cardiorespiratory fitness in critically ill pediatric patients^19^, HIV-infected children^20^. In healthy adolescents, the PhA was directly associated with the physical fitness composite z score in both sexes (male: β = 0.09, *p* < 0.01; female: β = 0.03, *p* = 0.05), therefore, directly associated with isolated and grouped physical fitness indicators for this age group. In this way, the PhA can be used to monitor the health of adolescents^20,21^, and healthy children^22^. However, few studies have linked physical fitness, body composition and PhA in adolescents. Furthermore, no correlational studies were found between PHA and allometric VO_2peak_ in adolescents. Thus, the aim of this study was to verify the association between levels of physical fitness, parameters of body composition and PhA. We hypothesized that adolescents who have higher PhA values will show better levels in body composition (i. e., > FFM; < FM) and in all physical fitness tests.

## Methods

### Participants and study design

This is a cross-sectional study with children of both sexes intentionally selected from a local school in the city of Campinas, Brazil. We recruited 358 children and following the inclusion criteria, they had to: a) be properly enrolled at the local school; b) detain a regular attendance at Physical Education classes, and c) be between 11 and 16 years old. From this total, children were excluded in the case of: a) presence of physical disabilities (permanent or temporary) that would prevent them from participating in any of the procedures (n = 6); b) use of prescribed medicine (n = 2); c) no completion of any of the measurements collected (n = 102); and d) not returning a signed Informed Consent Form (n = 96). Thus, 152 children (84 girls and 68 boys) were included in the data analysis of this study.

This research was approved by the Pontifical Catholic University of Campinas’ Research Ethics Committee by means of CAAE: 24727119.1.0000.5481. All procedures were conducted and followed the Declaration of Helsinki for studies with human subjects. Informed consent was obtained from all participants and their parents/guardians involved in the study. Data collection dates ranged from February 1 to April 30, 2018.

### Study design, tasks and procedures

All adolescents and their guardians/parents who agreed to participate in the study were adequately informed about the research proposal and the procedures to which they would be subjected. After obtained the parents’ consent, all tests were applied during the Physical Education (PE) classes and each class lasted for approximately 90 min in two different days. For each participant, all the measurements were obtained in the morning, after an overnight regular fast (8 h), refraining from vigorous exercise for at least 15 h, avoiding caffeine and alcohol during the preceding 24 h, and consuming a normal evening meal the night before. Verbal explanation and test demonstration were performed before participants were tested. The evaluation was done by properly trained professionals.

#### Anthropometry assessment

Anthropometry and body composition^23^ were measured before the physical fitness testing. Total body weight (kg) was determined using a digital scale (Sanny Digital Glass 200 Control, SBC, SP, Brazil) to the nearest 0.1 kg, and total body height (cm) using a vertical stadiometer (Sanny, SBC, SP, Brazil) to the nearest 0.1 cm. Consecutively, the body mass index (BMI: kg/m^2^) was calculated.

#### Maturity status

Maturity status was estimated using two approaches: sexual maturation, using self-examination of pubertal development for which the participants were provided with a standardized series of realistic color images with an explanatory text to individually assess their pubertal development following the sexual maturity stage criteria described by Tanner^24^. All assessments were carried out in a private room. Eventually, somatic maturation was verified, using estimations based on maturity offset and age at peak height velocity (PHV). The total age (years) and total body height (cm) were used to predict the PHV in years, following the published equation^25^:

$${\text{PHV}}_{{{\text{boys}}}} = \, - {7}.{999994 } + \, \left( {0.00{36124 } \times \, \left( {{\text{age }} \times {\text{ height}}} \right)} \right);{\text{ R}}^{{2}} = \, 0.{\text{896 and SEE }} = \, 0.{542}$$

$${\text{PHV}}_{{{\text{girls}}}} = \, - {7}.{7}0{9133 } + \, \left( {0.00{42232 } \times \left( {{\text{age }} \times {\text{height}}} \right)} \right);{\text{ R}}^{{2}} = \, 0.{\text{898 and SEE }} = \, 0.{528}$$

According to sex, a negative value of PHV was considered pre-PHV, while a positive value of PHV was considered post-PHV.

#### Body composition and phase angle assessment

BIA measurements were performed according to the protocol, using a Quantum II, single frequency (50 kHz) tetrapolar device (RJL Systems, Detroit, MI, USA). All children were instructed to remove all objects containing metal before BIA measurement was taken. Participants were laid barefoot, in a supine position, with the legs abducted at a 45° angle, arms far from the trunk and hands pronated on a table isolated from electrical conductors. After five minutes resting, the skin of the participants was cleaned with alcohol and two electrodes were placed on the surface of the right hand and two others on the surface of the right foot, according to the recommended protocol^14^. The evaluation lasted approximately one minute.

BIA provided values of R and Xc in Ω and, from these variables, PhA was calculated using the following published equation:^23^

$$PhA = {\text{arctangent}}\left( \frac{Xc}{R} \right) \times \left( {\frac{180}{\pi }} \right)$$

FFM was calculated using the published equation, by Houtkooper et al.^27^:

$${\text{FFM }} = \, 0.{61 } \times \, \left[ {{\text{height}}\left( {{\text{cm}}^{{2}} } \right) \, /{\text{ R}}} \right] \, + \, 0.{25 } \times {\text{ weight }} + { 1}.{31}$$

Then, the percentage of fat mass (%FM) was calculated:

$$\% {\text{FM }} = \, \left[ {\left( {{\text{body weight }}{-}{\text{ FFM}}} \right)/{\text{body weight}} \times {1}00} \right).$$

#### Physical fitness assessment

The cardiorespiratory fitness (VO_2peak_) was determined using Léger’s 20 m shuttle run test^28^ and, afterwards, was adjusted allometrically by weight, height and FFM according to the following equation:

$${\text{Log}}Y = {\text{ Log }}\alpha \, + k \cdot {\text{Log}}X$$

The coefficients observed for weight, height and FFM in girls were − 0.79, − 0.60, and − 0.84, respectively, while in boys were − 0.80, − 0.61, and − 0.87, respectively.

Neuromotor tests followed the procedures described by Silva et al.^29^ *Hamstring* and lumbar spine flexibility were measured by the sit-and-reach test using the Wells’ bench with feet supported at the 23-cm mark. Abdominal muscular endurance was assessed by the 1-min sit-ups test. Lower limb muscle explosive strength was evaluated by standing long jump test (SLJ). To evaluate upper limb explosive strength, the medicine ball throw (MBT) test was performed, using a 2 kg ball. Linear speed was evaluated by a 20-m sprint test (20SRT), and agility was evaluated by 4-m shuttle run test (4SRT). Before each test, the test objectives and procedures were briefly explained to facilitate their understanding by the participant. Additionally, participants completed a pre-test before all physical fitness tests.

### Ethics approval and consent to participate

This study was approved by the Pontifical Catholic University of Campinas’ Research Ethics Committee by means of CAAE: 24727119.1.0000.5481. All procedures were conducted and followed the Declaration of Helsinki for studies with human subjects. Informed consent was obtained from all participants and their parents/guardians involved in the study.

### Statistical analysis

Sample size Post hoc analysis of the total sample was used to establish the power of the study at 0.99 (1 − β), with an effect size of 0.35 (critical F 2.276; *p* < 0.05), for the boys sample, the power of the study was 0.96 with an effect size of 0.35 (critical F 2.363; *p* < 0.05) and for the sample of girls, the power of the study was 0.99 with the size of effect of 0.35 (critical F 2.331; *p* < 0.05). The G × Power 3.1.9.7 software was used.

The statistical analysis was performed using SPSS software (version 25.0, Chicago, IL, EUA). Data normality was verified using Kolmogorov–Smirnov test and subsequently presented as mean and standard deviation (SD). Student’s *t*-test was employed to verify the differences between groups (boys and girls) for age, PHV and age of PHV variables. Pearson’s correlation was used to establish an association between PhA and anthropometric variables, body composition and physical fitness. Additionally, to determine differences of values of anthropometric variables, body composition and PhA between groups, Generalized Linear Models (GzLM) were implemented and adjusted by PHV and Bonferroni post hoc test. GzLM also allowed this study to evaluate the discrepancy between groups regarding the physical fitness test (except for allometric VO_2peak_), and PHV, height, FFM and %FM were adopted as covariables. Finally, to verify the determining factors of PhA (dependent variable), linear regression analysis of variation (stepwise method) was used, considering as independent variables those that showed a correlation with PhA for each sex (Table 3), the multicollinearity was determined by the Variance Inflation Factor (VIF < 5). Thus, the variables that presented greater explanatory power and statistical significance were included in the model. The level of significance adopted was *p* < 0.05.

## Results

The descriptive anthropometrics and body composition characteristics of the participants included in the study are presented in Table 1. As we found a difference of PHV between sex groups, the anthropometric variables, body composition, and physical fitness (with the exception of allometric VO_2peak_) were adjusted by PHV.

**Table 1:** Descriptive characteristics of the study participants stratified by sex.

Variables	Girls (n = 84)	Boys (n = 68)	*p*-Value
	Mean ± SD	Mean ± SD	
Age (years)	13.22 ± 1.22	13.25 ± 1.05	0.873*
PHV (years)	0.85 ± 1.10	− 0.58 ± 0.86	< 0.001*
Height (m)	1.49 ± 0.06	1.61 ± 0.08	**< 0.001**^**#**^
Weight (kg)	44.02 ± 1.18	53.15 ± 1.53	**< 0.001**^**#**^
BMI (kg/m^2^)	19.58 ± 0.43	20.37 ± 0.56	0.266^**#**^
BMI (z escore)	0.41 ± 1.25	0.23 ± 1.08	0.372^**#**^
Resistance (Ω)	690.16 ± 9.38	575.74 ± 12.23	**< 0.001**^**#**^
Reactance (Ω)	64.91 ± 0.89	61.72 ± 1.16	**0.029**^**#**^
FFM (kg)	32.42 ± 0.68	42.75 ± 0.88	**< 0.001**^**#**^
FM (%)	25.24 ± 0.79	19.12 ± 1.04	**< 0.001**^**#**^
Phase Angle (°)	5.43 ± 0.08	6.20 ± 0.11	**< 0.001**^**#**^

*PHV* Peak height velocity, *BMI* Body mass index, *FFM* Fat-free mass, *FM,* Fat mass.

*Student’s *t*-test.

^#^Generalized Linear Model (adjusted by PHV). Bold values indicate statistically significant (boys vs. girls, *p* < 0.05).

In this study, girls had higher %FM and lower body weight, height and FFM compared to boys. They also had higher values of R and Xc, and had lower PhA values compared to boys (Table 1).

The association of PhA and PHV according to girls and boys established that girls’ PhA was not associate with PHV (r = 0.16; *p* = 0.138), whereas for boys, an association was found (r = 0.28; *p* = 0.019); for this reason, controlled correlation by PHV (with the exception of allometric VO_2peak_) was used in the analysis for boys.

PhA in girls was not associated with allometric VO_2peak_ FFM, allometric VO_2peak_ weight, allometric VO_2peak_ height, or with 4SRT and 20SRT. However, an association between PhA and weight, FFM, BMI, %FM and MBT was found for girls. PhA was not associated either with %FM in boys. However, an association between PhA and weight, FFM, BMI, SLJ, MBT, allometric VO_2peak_ FFM, allometric VO_2peak_ weight, and allometric VO_2peak_ height was found in boys. In addition, a negative association was found between PhA and 4SRT and 20SRT in boys (Table 2).

**Table 2:** Correlation analysis between phase angle and determinant factors in the study population stratified by sex.

Variables	Girls (n = 84)		Boys (n = 68)	
	r	*p*	r	*p*
Height (m)	0.067	0.544	0.106	0.393
Weight (kg)	0.432	< 0.001	0.359	0.003
BMI (kg/m^2^)	0.524	< 0.001	0.352	0.004
FFM (kg)	0.448	< 0.001	0.486	0.000
FM (%)	0.377	< 0.001	0.072	0.561
Flexibility (cm)	0.266	0.014	0.117	0.346
Abdominal (repetitions)	− 0.010	0.927	0.297	0.015
SLJ (cm)	− 0.016	0.884	0.341	0.005
MBT (cm)	0.357	0.001	0.600	0.000
4SRT (s)	− 0.028	0.798	− 0.245	0.046
20SRT (s)	− 0.029	0.791	− 0.297	0.015
VO_2_ peak (ml^−1^/kg^−1^/min^−1^)	− 0.121	0.273	0.243	0.046
VO_2_ peak (ml/weight/min)	0.158	0.151	0.398	0.001
VO_2_ peak (ml/height/min)	− 0.118	0.286	0.259	0.033
VO_2_ peak (ml/FFM/min)	0.126	0.252	0.406	0.001

*BMI* Body mass index, *FFM* Fat-free mass, *FM* Fat mass, *SLJ* Standing long jump, *MBT* Medicine ball throw test, *4SRT* 4-m shuttle run test, *20SRT* 20-m sprint test.

*Pearson Correlation (adjusted by PHV).

Table 3 presents the physical fitness test stratified by sexes. The results (with the exception of allometric VO_2peak_) were adjusted for the confounding variables (PHV, height, FFM, and %FM). With the exception of flexibility test (girls: 24.30 ± 0.94 cm vs. boys: 18.24 ± 1.24 cm, *p* < 0.001), boys showed higher values in all physical tests compared to girls (*p* < 0.001).

**Table 3:** Physical fitness test of the study participants stratified by sex.

Physical test	Girls (n = 84)	Boys (n = 68)	*p*
	Mean ± SD	Mean ± SD	
Flexibility (cm)	24.30 ± 0.94	18.24 ± 1.24	< 0.001^**#**^
Abdominal (repetitions)	24.64 ± 1.00	35.86 ± 1.31	< 0.001^**#**^
SLJ (cm)	129.61 ± 2.50	172.91 ± 3.26	< 0.001^**#**^
MBT (cm)	245.00 ± 5.70	368.18 ± 7.41	< 0.001^**#**^
4SRT (s)	7.14 ± 0.80	5.56 ± 0.10	< 0.001^**#**^
20SRT (s)	4.41 ± 0.05	3.87 ± 0.07	< 0.001^**#**^
VO_2_ peak (ml^-1^/kg^-1^/min^-1^)	39.88 ± 3.20	43.45 ± 4.78	< 0.001*
VO_2_ peak (ml/weight/min)	115.08 ± 8.29	127.79 ± 14.29	< 0.001*
VO_2_ peak (ml/height/min)	105.09 ± 8.26	116.55 ± 12.89	< 0.001*
VO_2_ peak (ml/FFM/min)	114.76 ± 8.75	133.43 ± 16.53	< 0.001*

*SLJ* Standing long jump, *MBT* Medicine ball throw test, *4SRT* 4-m shuttle run test, *20SRT* 20-m sprint test.

*Student’s *t*-test.

^#^Generalized Linear Model (adjusted by PHV, height, FFM, and %FM).

Table 4 shows the linear regression analysis using the stepwise method between PhA, body composition, and physical fitness test in the study participants stratified by sex.

**Table 4:** Linear regression analysis between phase angle and determinant factors in the study population stratified by sex.

Models		B	EP	R^2^	β	t	*p*	95%CI	
								IL	UL
GIRLS (n = 84)									
1	(Constant)	− 0.036	0.094			− 0.383	0.703	− 0.222	0.150
	BMI	0.522	0.097	0.259	0.509	5.360	0.000	0.329	0.716
2	(Constant)	− 0.016	0.090			− 0.174	0.862	− 0.195	0.164
	BMI	0.849	0.150	0.324	0.827	5.647	0.000	0.550	1.148
	% FM	− 0.439	0.158		− 0.407	− 2.777	0.007	− 0.753	− 0.124
BOYS (n = 68)									
1	(Constant)	− 0.086	0.122			− 0.702	0.485	− 0.329	0.158
	Height	0.316	0.139	0.073	0.270	2.274	0.026	0.039	0.594
2	(Constant)	− 0.137	0.099			− 1.385	0.171	− 0.334	0.060
	Height	− 0.653	0.196	0.406	− 0.557	− 3.336	0.001	− 1.045	− 0.262
	FFM	1.275	0.211		1.008	6.039	0.000	0.853	1.696
3	(Constant)	0.024	0.095			0.252	0.802	− 0.166	0.214
	Height	− 0.751	0.175	0.538	− 0.640	− 4.277	0.000	− 1.101	− 0.400
	FFM	0.728	0.227		0.576	3.209	0.002	0.275	1.181
	MBT	0.621	0.145		0.620	4.283	0.000	0.331	0.910
4	(Constant)	− 0.003	0.092			− 0.029	0.977	− 0.186	0.181
	Height	− 0.739	0.168	0.584	− 0.630	− 4.399	0.000	− 1.074	− 0.403
	FFM	0.823	0.220		0.651	3.742	0.000	0.384	1.263
	MBT	0.527	0.143		0.527	3.686	0.000	0.242	0.813
	4SRT	− 0.221	0.084		− 0.221	− 2.636	0.011	− 0.389	− 0.053

*BMI* body mass index, *FFM* Fat-free mass, % *FM* Fat Mass percentage, *MBT* Medicine ball throw test, *4SRT* 4-m shuttle run test, 95%*CI* confidence interval 95%, *IL* inferior limit, *UL* upper limit.

Overall, the greater predictor for girls is BMI with explanatory significance of 25.9% (*p* < 0.001), succeeded by %FM with 6.5% (*p* = 0.007). Concurrently, FFM was the main predictor for boys, demonstrating 33.3% (*p* < 0.001) of PhA variation, succeeded by MBT with 13.2% (*p* < 0.001), height with 7.3% (*p* = 0.001), and 4SRT with 4.6% (*p* = 0.011).

## Discussion

This study evaluated the association between PhA, body composition, and physical fitness tests among a sample of adolescent boys and girls. To date, no studies have been found that verify the association of PhA with indicators of physical fitness, mainly allometric VO_2peak_ in adolescents in this age group. We established that boys showed a higher PhA value than girls (6.20 ± 0.1 and 5.43 ± 0.08; *p* < 0.001, respectively). Furthermore, the study established a positive correlation between PhA and the body composition variables, FFM for both sexes, and FM and BMI in girls, as a higher FFM value was clearly associated with a higher PhA. Overall, FFM was also identified as the main predictor for boys, demonstrating 33.3% of PhA variability, whereas for girls, BMI was the greatest predictor, with explanatory significance of 25.9%.

The results of this study establish higher values for girls in relations to boys for PHV, R, Xc, and %FM, although boys depicted greater values for weight, height, FFM, and PhA, reinforcing outcomes documented in other studies with scholar adolescents^30^.

Moraes et al.^31^ showed that variation in maturity status significantly influenced the PhA of female and male adolescents aged between 10 and 15 years, adjusting for variation associated with sex and age. And within each age group, adolescents with more advanced stages of pubic hair, especially those at the end of puberty and mature, had higher PhA values. However, maturity- and age-associated variation in PhA was significantly accounted for when partitioning for body mass. Thus, the influence of body mass appears to mediate the variation associated with maturity and age in PhA in adolescents, regardless of sex.

Interestingly, the study reported superior PhA values for boys rather than girls, which could be explained by the fact that boys present greater FFM and lower %FM than girls, being well established that FFM is an exceptional electrical conductor^14^. This finding was consistent with a previous review by Mattiello et al.^32^, which evaluated 46 studies including 249.844 subjects and reported a 7.3° value of PhA (95%CI 7.0° and 7.5°) for adolescent (16–18 years old) boys and 6.4° (95%CI 6.1° and 6.8°) for girls.

Weak and moderate correlation was observed for both sexes between PhA and weight, FFM, MBT, and BMI, exclusively for girls in FM and %FM, and primarily for boys regarding SLJ, 4SRT, 20SRT, and the three allometric VO_2peak_ considered. Furthermore, in girls, the predictors were the body composition variables associated with fat increase (BMI and %FM), while in boys, the predictors were the variables related to the neuromuscular growth (i.e., height, FFM, MBT, and 4SRT); nonetheless, the study was unable to establish correlations with physical fitness tests (i.e., flexibility and VO_2peak_ ml^−1^/kg^−1^/min^−1^). It is imperative to display the negative impact of %FM for girls and 4SRT for boys; however, this variable is measured in seconds (i.e., time), which represents that the smaller the time, the better the results of the test. Hence, even with negative results found in linear regression analysis (*B* = − 0.439)*,* there is a positive influence on PhA. These data emphasize the discrepancy during growth induced by puberty in both sexes and can be clarified as girls experience puberty earlier than boys. Additionally, female puberty is modulated by estrogen, which enhances the build-up of fat; meantime masculine puberty modulated by androgen increases the development of fat-free mass^5^.

PhA index reported in this study were within the normality standard for this age group, which outlines a progressive increase during adolescence. In their research, Langer et al.^30^ have shown that physical training is associated with higher PhA values and thus improving cellular health. Besides, superior PhA values (6.9° ± 0.9°) were obtained by adolescents who practice sports, compared to adolescents who did not exercise.

PhA index represents the cellular function as it is composed by the relationship established between R and Xc. Such relationship is important because it reflects different electrical 14properties of tissues that can be affected in various ways by diseases, nutritional status and hydration status^14^. Overall, when a subject produces a force, the individual generates an electrical current throughout the neurons that transport it continuously to the muscle, which endure low resistance to the transmission of the current, being highly conductive and generating the muscular contraction that modifies the existing relation between the electrolytes confined in the water of the tissue, therefore, altering the values of R^33^, and consequently PhA value.

Regarding girls in this study, BMI and %FM account for 32.4% of PhA variation, while for boys, height, FFM, MBT, and 4SRT have an explanatory significance of 58.4% on PhA variation. Boys’ results endorse previous observational studies that validated a direct correlation of PhA and diverse functional indexes, such as handgrip strength, knee extension strength, and maximum quadriceps strength^20^.

It is known that after early adolescence, boys experience a greater increase in the development of muscular strength compared to girls^7^, which may justify the differences in physical performance between the sexes at this stage. Furthermore, it was demonstrated in the previous study^34^ that the chest medicine ball throw and vertical jump are excellent predictors of maximum power and muscular power and the MBT prove to be a good predictor, even after controlling for sex, age, height, weight and maturation. As observed in the present study, boys showed better results in lean mass, which, in turn, is made up in a greater percentage of muscle mass, the main predictor of strength and power and also an excellent conductor of electricity due to its high levels of water body^20^. This may partly explain the association of the MBT and SLJ tests only in males. PhA is inversely proportional to resistance (Ω), which depends on intra and extracellular water levels, in this sense, muscle mass is directly related to muscular strength^21^. Furthermore, the social and cultural context [(i.e. girls participate in fewer physical activities (moderate and vigorous intensities) and sports than boys], may reflect the lower performance in physical tests in girls, contrary to the better motor gestures in boys^35^.

Gonzalez et al.^36^ report in their research that age was the predicting leading factor for PhA in men and women, succeeded by FFM and height. FFM is a good electrical conductor because it has a greater amount of water, thus a greater amount of electrolytes, interfering directly in PhA, which is measured according to the relationship between R and Xc. As formerly mentioned, R is inversely related to the amount of electrolytes enclosed in the water of the tissue; therefore, if the tissue hydration level is low, R values increase, whereas if it is high, R will be smaller^14^. Although Xc indicates the capacitance of the cellular membrane, as it is a non-ionic tissue surface, it prolongs the transmission of electrical flow, thus, the better the integrity of the cellular membrane, the higher the body’s Xc values.

PhA is widely used in numerous populations as an indicator of nutritional status and cellular function^33^, in which elevated PhA values in healthy individuals suggest a better cellular function^37^, indicating a larger cellular body mass and a greater volume of intact cellular membrane. On the other hand, low PhA values are found in subjects carrying diseases, such as patients with anorexia nervosa, and HIV^20^ and cancer patient^37,38^, thus revealing the impairment of the cellular function and integrity of the membrane.

This study is important because it uses the 20SRT result in VO_2max_, and not in number of laps. This allowed the calculation of allometric VO_2_, therefore, the adjustment of oxygen consumption (ml.min.) by body mass (ml.kg.min.^-1^) is insufficient (linear relationship), as the changes that occur in body dimensions over time, in general, are both structural and functional. Thus, the allometric scale assumes that the relationship between VO_2_ and body mass changes in children and adolescents at different maturational stages (i.e. curvilinear)^39^. Therefore, it also contributes to providing a reference for the exponent “*b*” for calculating the allometric VO_2_, taking into account the weight, height and FFM.

Furthermore, we use easily applicable strength tests, at low cost and with materials available in the school environment, mainly in developing countries, such as the MBT and SLJ (i.e. medicine ball balls, measuring tape and/or or measuring tape), instead of more sophisticated and expensive materials.

The limitations of this study are due to its transversal nature that impedes a cause-effect relationship validation, and the small group of participants that prevents results from being extrapolated to other population groups. For girls, data on the first menarche were not obtained. Furthermore, information about physical activity and exercise that could increase the precision of our main exposures were not collected. Additionally, we included participants of any nutritional status. Furthermore, the suggestion is the performance of future longitudinal studies to better comprehend the cause-effect relationship between PhA, body composition, and physical fitness.

By this time, no other study has used seven tests (i.e., flexibility, abdominal, SLJ, MBT, 4SRT, 20SRT and VO_2peak_) to determine the association between physical fitness and PhA with this number of healthy adolescents. This study verified that PhA is a health marker that presents variability according to the levels of physical fitness and body composition of adolescents.

## Conclusion

In this sample, boys showed greater PhA values compared to girls. We found a positive association between PhA and FM for girls and between PhA and FFM for boys. In addition, physical fitness tests (flexibility, abdominal, SLJ, MBT, 4SRT, 20SRT, and VO_2_ peak) showed an association with PhA. Therefore, PhA can be implemented as critical tool to assess cellular health and monitor the nutritional status, overall health in children and adolescents. Therefore, PhA can be implemented as a critical tool to assess cellular health and monitor nutritional status, used in physical assessments and screening of physical activity recommendations related to the general health of children and adolescents.

## Data Availability

The dataset used and analyzed during the current study are available from the corresponding author on reasonable request.
